# Clitocine Reversal of P-Glycoprotein Associated Multi-Drug Resistance through Down-Regulation of Transcription Factor NF-κB in R-HepG2 Cell Line

**DOI:** 10.1371/journal.pone.0040720

**Published:** 2012-08-22

**Authors:** Jianguo Sun, Chilam Au Yeung, Ngai Na Co, Tsun Yee Tsang, Esmond Yau, Kewang Luo, Ping Wu, Judy Chan Yuet Wa, Kwok-Pui Fung, Tim-Tak Kwok, Feiyan Liu

**Affiliations:** 1 Zhejiang University, Research Centre of Siyuan Natural Pharmacy and Biotoxicology, College of Life Sciences, Zijinggang Campus, Hangzhou, People's Republic of China; 2 Zhejiang University, Joint centre of Zhejiang University and The Chinese University of Hong Kong on Natural Products and Toxicology Research, Zijinggang Campus, Hangzhou , People's Republic of China; 3 School of Biomedical Sciences (SBS), The Chinese University of Hong Kong, Shatin, Hong Kong SAR, People's Republic of China; Bauer Research Foundation, United States of America

## Abstract

Multidrug resistance(MDR)is one of the major reasons for failure in cancer chemotherapy and its suppression may increase the efficacy of therapy. The human multidrug resistance 1 (MDR1) gene encodes the plasma membrane P-glycoprotein (P-gp) that pumps various anti-cancer agents out of the cancer cell. R-HepG2 and MES-SA/Dx5 cells are doxorubicin induced P-gp over-expressed MDR sublines of human hepatocellular carcinoma HepG2 cells and human uterine carcinoma MES-SA cells respectively. Herein, we observed that clitocine, a natural compound extracted from *Leucopaxillus giganteus*, presented similar cytotoxicity in multidrug resistant cell lines compared with their parental cell lines and significantly suppressed the expression of P-gp in R-HepG2 and MES-SA/Dx5 cells. Further study showed that the clitocine increased the sensitivity and intracellular accumulation of doxorubicin in R-HepG2 cells accompanying down-regulated MDR1 mRNA level and promoter activity, indicating the reversal effect of MDR by clitocine. A 5′-serial truncation analysis of the MDR1 promoter defined a region from position −450 to −193 to be critical for clitocine suppression of MDR1. Mutation of a consensus NF-κB binding site in the defined region and overexpression of NF-κB p65 could offset the suppression effect of clitocine on MDR1 promoter. By immunohistochemistry, clitocine was confirmed to suppress the protein levels of both P-gp and NF-κB p65 in R-HepG2 cells and tumors. Clitocine also inhibited the expression of NF-κB p65 in MES-SA/Dx5. More importantly, clitocine could suppress the NF-κB activation even in presence of doxorubicin. Taken together; our results suggested that clitocine could reverse P-gp associated MDR via down-regulation of NF-κB.

## Introduction

Cancer cells can develop resistance against structurally and mechanistically unrelated chemotherapeutic agents, a phenomenon named as multidrug resistance (MDR) [1]. Although numerous resistant mechanisms are known, a large number of evidence strongly supports the important role of energy-dependent efflux systems (e.g. P-glycoprotein (P-gp)) that pump anti-cancer agents out of the cells [2]. P-gp is a 170 kDa protein that belongs to the ATP-binding cassette (ABC) superfamily of membrane transporter proteins and is encoded by the MDR1 gene [3], [4].

Since P-gp was first identified three decades ago, its structure and function have been extensively characterized. However, time has clearly demonstrated that P-gp induced multidrug resistance is much more complex than initially supposed. Until recently, scientists have just got the high resolution x-ray structure of mouse P-gp but not human's [5]. Efforts to circumvent the P-gp associated MDR in clinic mainly focus on the use of modulators which block the P-gp mediated efflux of anticancer agents. In general, P-gp can be inhibited by: i) blocking the drug-binding site(s) either competitively, non-competitively or allosterically; ii) interfering ATP hydrolysis and iii) altering the integrity of cell membrane lipids [6]. A large number of chemical agents including calcium blocker, calmodulin inhibitors, coronary vasolilators, indole alkaloids, quinolines, hormones, cyclosporins, surfactants and antidodies are modulators which could reverse the pump function of P-gp [7]. For example, the calcium channel blocker verapamil has been well-known to reverse multidrug resistance by directly binding to P-gp protein on special sites [8], [9]. Cyclosporine such as cyclosporin A could modulate the efflux function of P-gp by interfering with both the substrate-binding sites and the ATP hydrolysis cycle [10]. However, most of these agents necessitated high doses and produced unacceptable toxicity because of their low affinity with P-gp. Although new generation of P-gp inhibitors with high affinity at very low doses, such as elacridar (GF120918), tariquidar (XR9576) and OC144-093 (ONT-093) have been developed, novel approaches in overcoming P-gp associated MDR are still needed.

P-gp is highly regulated, especially at the transcriptional level. This indicates a promising approach in blocking of MDR, by inactivation of P-gp expression than by blocking its function. Although the mechanism for transcription regulation of MDR1 is still not fully understood, a large number of transcription factors such as Ras [11], Sp1 [12], p53 [13], NF-κB [14] and PKC [15] were reported to be involved. Some extracellular stimuli such as heat shock and chemotherapeutic agents could induce mdr1 mRNA expression [16], [17]. Fujita, T. et al indicated that paclitaxel induced nuclear translocation of YB-1 followed by increased expression of MDR1 in MCF-7 cells [18]. Recently, it is reported that DNA methylation and histone acetylation may be involved in the MDR1 regulation by changing chromatin configuration [19], [20]. For example, histone deacetylase (HDAC) inhibitors treatment leads to an increase in MDR1 expression through dynamic changes in chromatin structure and transcription factor association within the promoter region [21].

Nature compounds are gaining increasing interest in cancer therapy. Some agents extracted from fruits, vegetables, oilseeds, and plant herbs were able to modulate the activity of P-gp [22], [23], [24]. Clitocine, a nucleoside firstly isolated from the mushroom *Clitocybe inversa* in 1980s, was found to be a substrate and inhibitor of adenosine kinase [25]. Recent years, clitocine was reported to exert an anti-tumor effect in varies cancer cell lines such as Hela and MCF-7 cells, and many derivatives of this compound were also synthesized and examined for their biological properties [25], [26], [27], [28]. In the present study, we found that clitocine reverse the P-gp associated multidrug resistance in cancer cells. Furthermore, clitocine inactivates MDR1 expression through down-reguation of NF-κB as demonstrated *in vitro* and *vivo*.

## Materials and Methods

### Drugs and antibodies

**Figure 1:**
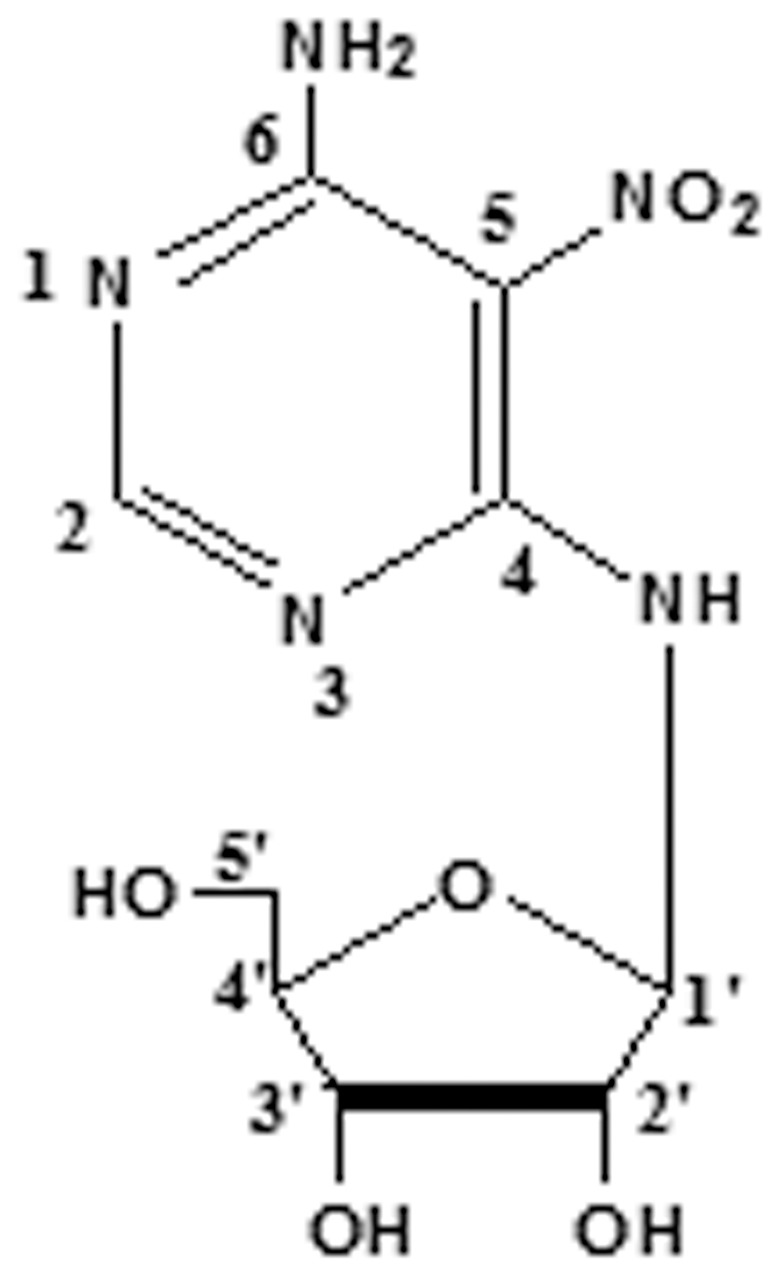
The structure of clitocine.

Clitocine was extracted from *Leucopaxillus giganteus* and its' chemical structure was presented in Fig. 1. Its' formula is C_9_H_13_N_5_O_6_ and molecular weight is 287. The purity of compound used in this study was >99%. Additionally, we also provided HPLC data (Figure S1) and NMR data (Table S1) of clitocine.

Doxorubicin (Dox) was purchased from Sigma Chemical Co. (St. Louis, MO). Bay 11-7082 was from Calbiochem (San Diago, CA). The antibodies mouse monoclonal anti-P-gp, rabbit polyclonal anti-NF-κB p65 were purchased from Cell Signaling (MA), mouse monoclonal anti-β-actin from Sigma (St. Louis, MO), horseradish peroxidase conjugated secondary antibodies from Santa Cruz, (CA). All other chemical reagents were from Sigma–Aldrich Chemical Co. (St. Louis, MO).

In all experiments, drugs were dissolved in DMSO as stock. The final concentrations of the drugs were prepared by diluting the stock with DMEM culture medium, with final concentration of less than 0.2% DMSO.

### Plasmids

The promoter sequences of human MDR1 gene were cloned by PCR, using genomic DNA purified from R-HepG2 cells as template, with the reverse primer 5′-CTCTAAGCTTGTCTCCAGCATCTCCACG-3′(+525) and the following forward primers: 5′-GAG AGCTAGCGAAAGTGGAAACATCCTCAG-3′(from−988), 5′- GAGAGCTAGCAAATGTTGGCAGTAAATATGGAA-3′(from−732), 5′-GAGAGCTAGCTGCTGAAGAAAGACCACTGC-3′(from−450), 5′-GAGAGCTAGCTAGAGAGGTGCAACGGAAGC-3′(from−193), 5′-GAGAGCTAGCCCGCTGTTCGTTTCCTTTAG-3′(from+44), and 5′-GAGAGCTAGCGGGACCAAGTGGGGTTAGAT-3′(from+270). The PCR products were digested with Hind II and Nhe I (sites underlined in the primers) and subcloned into pGL3 [*luc+*] Basic Vector (Promega). The open reading frame sequence of human NF-κB p65 (RELA) was cloned by PCR using total cDNA synthesized from R-HepG2 cells as template, with the forward primers: 5′- CCCAAGCTTATGGACGAACTGTTCCCC -3′and the reverse primer 5′- CCGCTCGAGTTAGGAGCTGATCTGACTC- 3′ .The PCR products were digested with HindIII and XhoI (sites underlined in the primers) and subcloned into pcDNA3.1. The putative NF-κB binding site on pGL3 (−988/+525) was mutated by site-directed mutagenesis using Quikchange II site-directed kit (Stratagene, La Jolla, CA) with forward primer 5′-TAAATGCGAATCCCGAGAAAA*TTT*CCCTTAACTACGTCC-3′ and reverse primer 5′-GGACGTAGTTAAGGG*AAA*TTTTCTCG GGATTCG CATTTA-3′; mutated nucleotides were indicated as italic letters. The integrity of the respective plasmid constructs was confirmed by DNA sequencing.

### Cell culture

Human hepatoma HepG2 and SMMC-7721 cells,human cervical cancer HeLa cells, human gastric cancer SGC-7901 cells, human uterine cancer MES-SA and MES-SA/Dx5 cells, human breast carcinoma MCF-7 and Bcap37 cells were obtained from American Type Culture Collection, were maintained in DMEM medium (Invitrogen, Inc., Carlsbad, CA) supplemented with 5% (v/v) fetal bovine serum (FBS), 2 mM L-glutamine, in 37°C humidified 10% CO2 incubator. Doxorubicin-induced multidrug resistant human hepatoma cells R- HepG2 was a kind gift provided by Prof. Kwok-pui Fung from the Chinese University of Hong Kong. To maintain the Dox-resistance, R-HepG2 and MES-SA/Dx5 cells were cultured with 1.2 µM Dox during passages. From time to time, the sensitivity of cells to Dox was analyzed for their resistance to cell death. Cells were confirmed as mycoplasma free using DAPI staining assay.

### Drug sensitivity assay

Cell survival after exposure to the anti-tumor agents was examined by MTT cytotoxicity assay. Cells were seeded in each well of a 96-well plate for 24 h. After incubation with various concentrations of doxorubicin, clitocine, or both for 48 h, the medium was discarded. Then, 50 µl of 1 mg/ml 3-(4,5-dimethylthiazol-2-yl)-2,5-diphenyltetrazolium bromide (MTT, Sigma–Aldrich Chemical) was added to each well for 4 h incubation at 37°C. The purple formazan formed was then solubilized by DMSO and absorbance at 570 nm was read by a microplate reader (Molecular Devices, Sunnyvale, CA). The data of MTT assay used clitocine or doxorubicin alone respectively and in combination in the absence of cell indicated that MTT reagents did not interfere with these two compounds (data not shown).

### Cellular doxorubicin accumulaton assay

Cells were seeded in 35 mm culture dish for 24 h to allow attachment. Then, the cells were incubated with 2 µM doxorubicin alone or together with 0.2 µM clitocine for 24 h. After being washed twice with PBS, the cells were measured by a FACSort flow cytometer (Becton Dickison) and then analyzed with CellQuest software.

### Western blot analysis

After incubation with clitocine for 48 h, the cells were lysed in lysis buffer (2.1 µg/ml aprotinin, 0.5 µg/ml leupeptin, 4.9 mM MgCl2, 1 mM orthovanadate, 1% Triton X 100, and 1 mM phenylmethylsulfonyl fluoride). Proteins in cell lysate were resolved in denaturing SDS-PAGE gel and transferred to Immobilon PVDF membrane (Millipore, Billerica, MA). After blocking with 5% nonfat dry milk, the membranes were washed by PBS containing 0.1% Tween 20 and incubated with primary antibodies followed by respective horseradish peroxidase conjugated secondary antibodies. Signals were visualized with enhanced ECL chemiluminescence detection reagents (Amersham Life Sciences, Inc., Buckinghamshire, United Kingdom) and visualized on X-ray film (Fuji Photo Film, Tokyo, Japan). Densitometric analysis of the protein bands was done using Image J (NIH). β-actin was used as reference to determine the protein relative level.

### Quantitative RT-PCR analysis

Total RNA was extracted by lyzing cells with TRI reagent (Molecular Research Center, Cincinnati, OH). Total RNA was used to synthesize the first strand cDNA by M-MLV reverse transcriptase (Promega, Madison, WI). Real-time PCR amplification was performed in ABI 7500 Fast Real-Time PCR system (Applied Bioscience, Foster City, CA) according to manufacturer's procedure for relative quantification. The amplification reactions were carried out with 1× Power SYBR Green PCR Master Mix (Applied Biosystems). PCR primers for MDR1 (forward, 5′-GCAGCTGGAAGACAAATACACAAA-3′; reverse, 5′-CCCCAACAT CGTGCACATC-3′), β-actin (forward, 5′-ACACCCCAGCCATGTACG TT-3′; reverse, 5′-TCACCGG AGTCCATCACGAT -3′) were designed by using the Primer Express 3.0 (Applied Biosystems). The standard temperature profile included initial denaturing at 95°C for 10 min, followed by 40 cycles of denaturing at 95°C for 15 s, annealing and extension at 60°C for 1 min. A DNA dissociation curve was generated to confirm the specificity of amplification. Relative Standard Cureve Method (2^−ΔΔCt^) was used to determine the relative mRNA expression using β-actin as reference. Validation experiment was carried out to ensure that amplification efficiency of the target genes and the reference gene was approximately equal.

### Luciferase reporter activity assay

R-HepG2 cells cells were seeded into 24-well plates one day before transfection. The cells were transfected with 1.2 µg of luciferase-reporter vector containing MDR1 promoter sequence using Lipofectamine 2000 (Invitrogen) and pGL3 empty vector was used as a negative control. 24 ng of pRL-CMV, which encoding Renilla luciferase, was included in all transfections to normalize transfection efficiency. 24 h after transfection, the cells were washed and lysed with the passive lysis buffer from the Dual-Luciferase Reporter Assay System (Promega). Luciferase activity was measured in each cell lysate using a FLUOstar Galaxy plate reader.

### Chromatin immunoprecipitation (CHIP) assay

ChIP assays were performed using the Chromatin Immunoprecipitation Assay kit based on the manufacturer's instruction (Upstate Group, Charlottesville, VA). Briefly, cells were fixed in 1% formaldehyde at 37°C for 10 min to cross-link histones to DNA. Cells were washed and detached from the dish by scraping following by addition of SDS lysis buffer. After 10 min incubation on ice, cells were sonicated to shear DNA. After centrifugation at 14,000 rpm for 10 min at 4°C, the sonicated cell supernatants were diluted with ChIP Dilution Buffer and aliquots of samples were saved as the input DNA for quantization of the amount of total DNA. For immunoprecipitation, 1 µg of NF-κB p65 antibodies or normal rabbit IgG was added to the precleared supernatants and incubated overnight at 4°C with rotation. Immunocomplexes were collected using Protein A Agarose/Salmon Sperm DNA for 1 h at 4°C. Following the wash, the immunocomplexes were recovered by resuspending in elution buffer at room temperature for 15 min. DNA-protein complexes as well as the input DNA were reverse cross-linked at 65°C for 4 h and treated with proteinase K at 45°C for 1 h. DNA was purified by phenol/chloroform extraction and ethanol precipitation. Thereafter, the DNA was subjected to PCR with primers: forward, 5′-TGCTGAAGAAAGACCACTGC-3′ and reverse, 5′- GCTTCCGTTGCACCTCTCT-3′. Amplification was carried out for 35 cycles with denaturation at 94°C for 30 s, annealing at 54°C for 30 s, and extension at 70°C for 40 s. PCR products were analyzed on a 1.5% agarose gel.

### Electrophoresis Mobility Shift Assay (EMSA) of NF-kB activation

NF-kB activation was analyzed using EMSA with NF-κB probe (AGT TGAGGGGACTTTCCCAGGC) as previously described [29]. Briefly, the Biotin-labeled probes were incubated with nuclear extracts for 60 min at room temperature. For specificity controls, unlabeled probe was added to the reaction at a 1∶100 molar excess. Anti-p65 subunit antibody (from Cell signaling) was also included to identify NF-κB-specific DNA binding. DNA-protein complexes were separated by electrophoresis in 5% polyacrylamide gels and signals were visualized with Luminol/Enhancer Solution detection reagents (thermo pierce) and visualized on X-ray film (Fuji Photo Film, Tokyo, Japan).

### Immunocytochemical staining method

R-HepG2 cells were seeded into 24-well plates for 24 h incubation. After incubation with 0.2 µM cliocine for 48 h, the cells were washed with PBS and fixed with 4% paraformaldehyde (PFA) in PBS for 10 min at room temperature. 100% ice-cold methanol was then used to permeablize the cells at −20°C for 10 min. After blocking with 3% BSA in PBS at room temperature for 45 min, the cells were incubated with anti-P-gp and anti- NF-κB p65 antibodies in blocking buffer for 2 h at room temperature and rinsed 3 times in PBS. The primary antibody was detected with goat anti-mouse IgG conjugated with FITC (for P-gp, Santa Cruz, CA) and goat anti rabbit antibody conjugated with RBITC (for NF-κB p65, Santa Cruz, CA). Nuclei were stained with DAPI (Roche). The levels of these proteins were observed with a LSM 510 fluorescence microscope fitted with appropriate filters and images were captured with an Orca II CCD camera (Hamamatsu, Bridgewater, NJ).

### Immunohistochemical staining method

R-HepG2 cells (1×10^7^ cells/mouse) were inoculated into nude mice and the tumors grew to up to appropriate size (i.e. tumor volume reached 100 mm^3^). Tumor bearing animals were divided into the two groups: intravenous injection of vehicle alone (control) or clitocine (10 mg/kg), each day for one week. Each group consisted of 5 animals. After 7 days, tumors were excised and fixed with 4% paraformaldehyde (PFA) in phosphate buffered saline (0.1 M PBS, pH 7.4) overnight. The tissues were rinsed three times in PBS, incubated overnight in 0.5 M sucrose in PBS, then embedded in Tissue-Tek® O.C.T. Compound, and cut into 6 µm frozen sections. The sections were washed with TBST for 15 min at room temperature and then blocked in 3% BSA in TBST (20 mM Tris, pH 7.5, 154 mM NaCl, 2 mM EGTA, 2 mM MgCl2, 0.1% Triton X-100) for 1 hour. Tissue sections were incubated with anti-P-gp and anti- NF-κB p65 antibodies in blocking buffer (3% BSA, 0.1% azide in TBST) for 1 h, then rinsed 3 times in TBST. The primary antibody was detected with goat anti-mouse IgG conjugated with FITC (for P-gp, Santa Cruz, CA) and goat anti Rabbit conjugated with RBITC (for NF-κB p65, Santa Cruz, CA). Nuclei were stained with DAPI (Roche). The levels of these proteins were observed with a LSM 510 fluorescence microscope fit with appropriate filters and images captured with an Orca II CCD camera (Hamamatsu, Bridgewater, NJ).

### Statistical analysis

In all experiments, data were expressed as mean ± standard deviation (SD). A significant difference of the sample's value from that of the respective controls in each experiment condition was assessed using Student's unpaired t-test with p value<0.05 being regarded as statistically significant.

## Results

### Anti-proliferation effect of clitocine on human cancer cell lines

The anti-proliferation effect of clitocine on human cancer cell lines was assessed by the MTT assay, including HepG2, R-HepG2, MES-SA, MES-SA/Dx5, SMMC7721, Bcap37, MCF-7, HeLa,and SGC-7901 for 48 h. The IC_50_ values were estimated as shown in Table 1. The treatment of the nine human cancer cell lines with clitocine exhibited a marked inhibition on the survival of these cells dose-dependently. Among them, drug resistant cells R-HepG2 and MES-SA/Dx5 showed similar sensitivity compared with their parental cells HepG2 and MES-SA.

**Table 1:** IC50 of clitocine for nine human cancer cell lines.

	Cell lines	aIC50(µM)
Cervical cancer	HeLa	14.9
Breast cancer	Bcap37	10.9
	MCF-7	43.0
	SMMC-7721	1.1
Liver cancer	HepG2	0.45
	R- HepG2	0.35
Gastric cancer	SGC-7901	2.2
Uterine cancer	MES-SA	0.62
	MES-SA/Dx5	0.7

a Cells (1×104) were incubated with various concentrations of clitocine for 48 h. Cell viability was measured by MTT assay. IC50 represents clitocine concentration causing a 50% growth inhibition.

### Reversal effect of clitocine in drug resistant cancer cells

**Figure 2:**
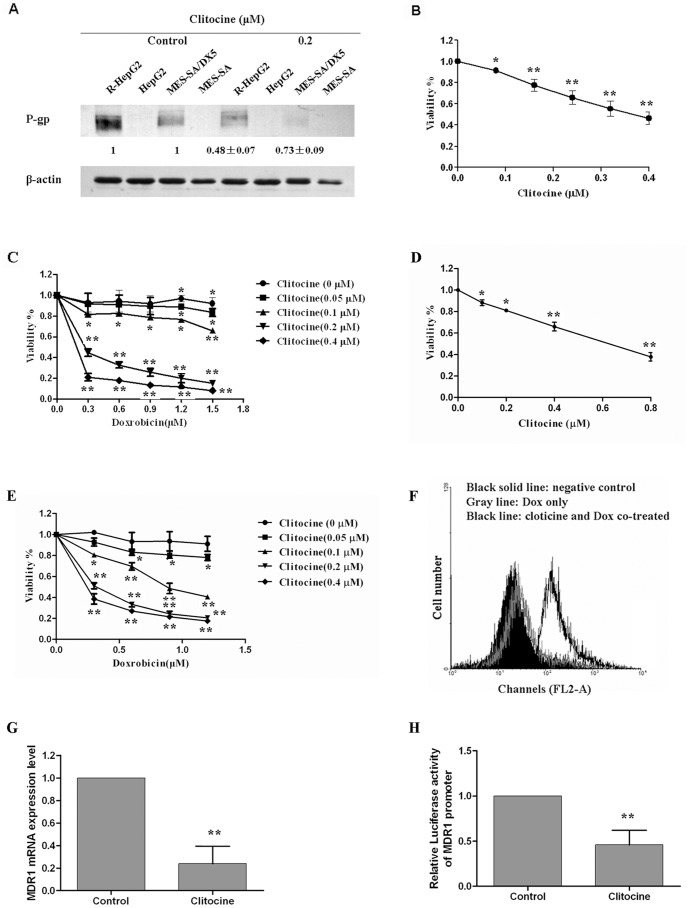
Reversal effect of clitocine in drug resistant cancer cells. (A) P-gp was detected in HepG2, R-HepG2, MES-SA and MES-SA/Dx5 cells by Western blot analysis as described in Materials and Methods. (B) After treatment with different concentrations of clitocine in R-HepG2 cells for 48 h, cell viability was determined by MTT assay. Data are mean ± SD, *N* = 6. (C) R-HepG2 cells were treated with different concentrations of doxorubicin together with 0, 0.05, 0.1, 0.2 or 0.4 µM clitocine respectively for 48 h. Cell viability was determined by MTT assay. Data are mean ± SD, *N* = 3. (D) After treatment with different concentrations of clitocine in MES-SA/Dx5 cells for 48 h, cell viability was determined by MTT assay. Data are mean ± SD, *N* = 3. (E) MES-SA/Dx5 cells were treated with different concentrations of doxorubicin together with 0, 0.05, 0.1, 0.2 or 0.4 µM clitocine respectively for 48 h. Cell viability was determined by MTT assay. Data are mean ± SD, *N* = 3. (F) Cellular doxorubicin accumulation level in R-HepG2 cells was measured by flow cytometry. The cells were incubated with 2 µM doxorubicin (Dox) alone or together with 0.2 µM clitocine for 24 h. The amount of doxorubicin accumulated in treated cells was quantified by flow cytometric method. (G) MDR1 mRNA level in R-HepG2 cells after clitocine treatment. After treatment with 0.2 µM clitocine for 48 h, total RNA in R-HepG2 cells was extracted. The MDR1 mRNA level in R-HepG2 cells was then measured by qRT-PCR analysis. Data are mean ± SD, *N* = 6. (H) The relative luciferase activity of full length MDR1 promoter reporter as determined by Dual-Luciferase Reporter Assay System. Cells were transiently transfected with full length MDR1 promoter reporter followed by treatment with 0.2 µM clitocine for 24 h. After that, the cells were lysed and the luciferase activity was measure. Data are mean ± SD, *N* = 6. ^*^ *P*<0.05 vs. control, ^**^ *P*<0.01 vs. control.

P-gp was reported to be over-expressed in the R-HepG2 [30] and MES-SA/Dx5 cells which was confirmed as in Fig. 2A. Surprisingly, clitocine can down-regulate the P-gp expression in both cell lines. More interestingly, clitocine can more effectively suppress the P-gp expression in R-HepG2 cells (about more than 50% at 0.2 µM). Although it seemed that higher level of P-gp was expressed in R-HepG2 cells than MES-SA/Dx5 cells.

Additionally, Clitocine could increase doxorubicin toxicity in R-HepG2 and MES-SA/Dx5 cells. As shown in Fig. 2B and D, clitocine exerted toxicity on R-HepG2 and MES-SA/Dx5cells with IC_50_ of about 0.35 µM and 0.7 µM respectively. To check whether clitocine may affect the sensitivity of drug resistant cancer cells to doxorubicin, the cells were treated with various concentrations of doxorubicin together with 0.05, 0.1, 0.2 or 0.4 µM clitocine respectively. As shown in Fig. 2C and E, co-incubation with ≥0.2 µM clitocine remarkably increased the response of R-HepG2 and MES-SA/Dx5 cells to doxorubicin. The synergistic effect of clitocine and doxorubicin was analyzed using the Chou-Talalay method [31]. As shown in Figure S2, combined treatment of clitocine and doxorubicin presented well as synergism (combination index; CI<1). Furthermore, clitocine also induced a detectable increase in cellular doxorubicin accumulation in R-HepG2 cells (Fig. 2F).

We next measured the MDR1 mRNA level in R-HepG2 cells with Real-time PCR analysis. As shown in Fig. 2G, the clitocine treatment significantly suppressed the mRNA level of MDR1. Using the dual-luciferase reporter assay, clitocine was also found to effectively down-regulate the activity of reporter with MDR1 promoter (Fig. 2H).

### Truncation analysis of MDR1 promoter sequence in relation to clitocine suppression effect

**Figure 3:**
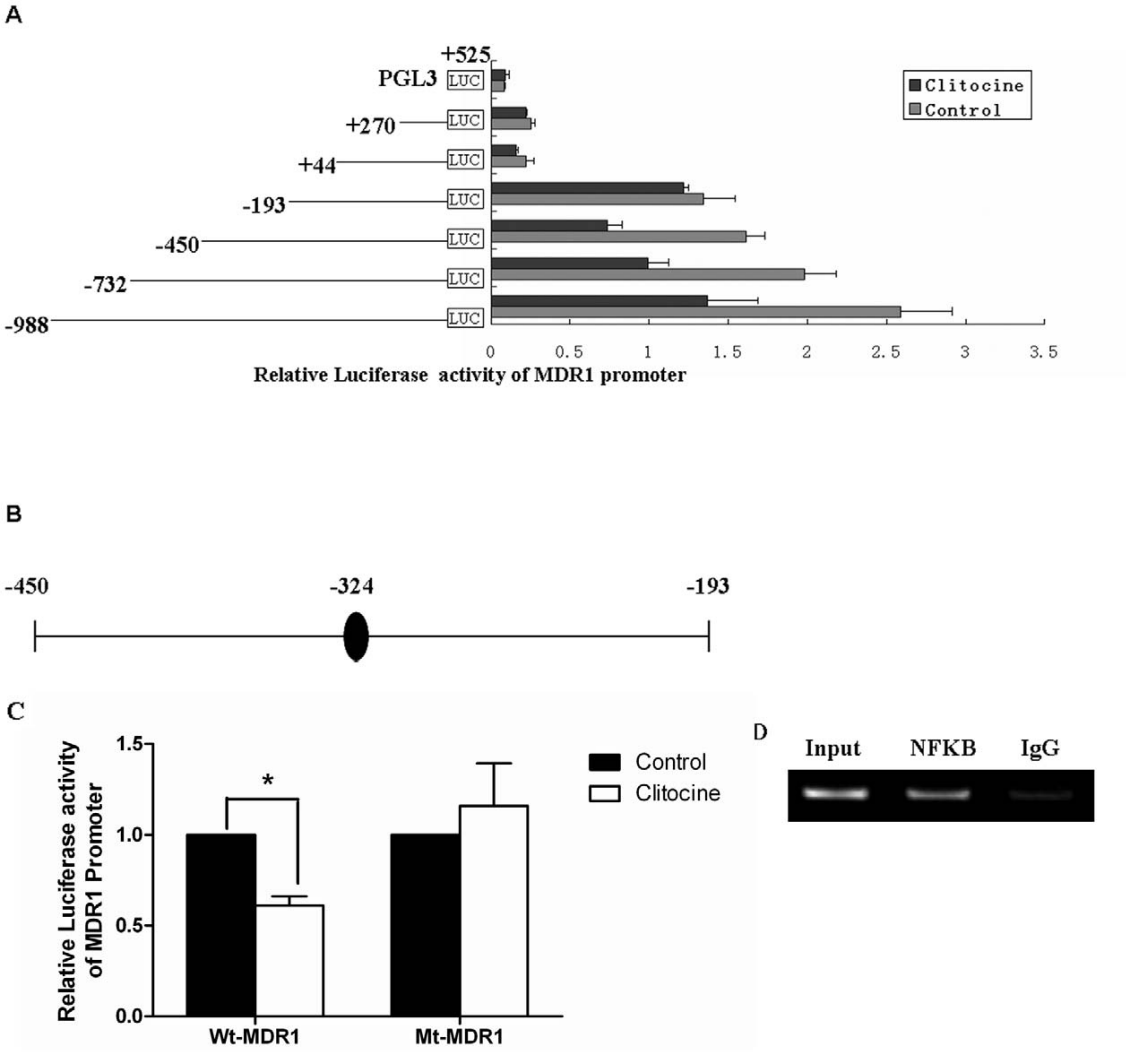
Truncation analysis of the MDR1 promoter with clitocine treatment. (A) The relative luciferase activity of 5′-end truncated MDR1 promoters was detected by Dual-Luciferase Reporter Assay System. R-HepG2 cells were transiently transfected with the truncated MDR1 promoter reporters followed by treatment with 0.2 µM clitocine for 24 h. After that, the cells were lysed and the luciferase activity was measured. Data are mean ± SD, *N* = 8. (B) Computational analysis of the putative transcription factor binding sites in the full length MDR1 promoter was performed. A single consensus binding site for transcription factor NF-κB was identified in the region −450 to −193. (C) R-HepG2 cells were transiently transfected with wild type (WT) and mutated type (MT) full length (−988∼+525) MDR1 promoter reporter followed by treatment with 0.2 µM clitocine in R-HepG2 cells for 24 h. After that, the cells were lysed and the luciferase activity was measured. Data are mean ± SD, *N* = 4. ^*^ *P*<0.05 vs. control. (D) Immunoprecipitaition (CHIP) assay was performed as described in Materials and Methods. Chromatin from R-HepG2 cells was cross-linked, sheard and immunoprecipitated with anti-NF-κB p65 antibody. Normal rabbit IgG was included as the negative control (IgG) and the input DNA from fragmented chromatin before immunoprecipitation was used as internal control. The recovered chromatin was subjected to PCR analysis using primers covering the putative NF-κB binding motif of the MDR1 promoter and the PCR products were resolved in 1.5% agarose gel. A representative experiment is shown, and similar results were obtained from three independent experiments.

In order to understand how MDR1 is suppressed by clitocine, a series of 5′-end truncated human MDR1 promoter fragments were cloned into the firefly luciferase reporter vector. These constructs were transiently transfected into R-HepG2 cells followed by incubation with 0.2 µM clitocine for 24 h. The luciferase activities were measured by dual reporter luciferase assay. As shown in Fig. 3A, clitocine remarkably decreased (by about 50%) the luciferase activities for the reporters with fragments −998/+525, −732/+525 and −450/+525 but showed no effect for those with fragments −193/+525, +44/+525 and +270/+525. The results indicated that the region between −193 and −450 may be responsible for the clitocine suppression effect on MDR1 promoter activity. Computational analysis for the putative transcription factor binding sites in the full length (−998/+525) MDR1 promoter sequence was performed using MATCH™ 1.0, MatInspector and Transcription Element Search Software (TESS). Interestingly, within the region −450 to −193, only one NF-κB putative binding site (−324 to −315) was identified by all three softwares (Fig. 3B). By mutating the NF-κB putative binding motif (−324 to −315), the inhibitory effect of clitocine on the activity of reporter with full length MDR1 promoter was significantly reversed (Fig. 3C). The results suggest that NF-κB may mediate the clitocine suppression on MDR1. Additionally, the binding of NF-κB to the putative binding site (−324 to −315) within the MDR1 promoter was confirmed by chromatin immunoprecipitaition (CHIP) assay. As shown in Fig. **3**D, a 277 bp PCR product was amplified from the DNA samples of R-HepG2 cells immunoprecipitated by anti-NF-κB p65 but not in the samples with normal rabbit IgG.

### NF-κB mediated the clitocine suppression on MDR1

**Figure 4:**
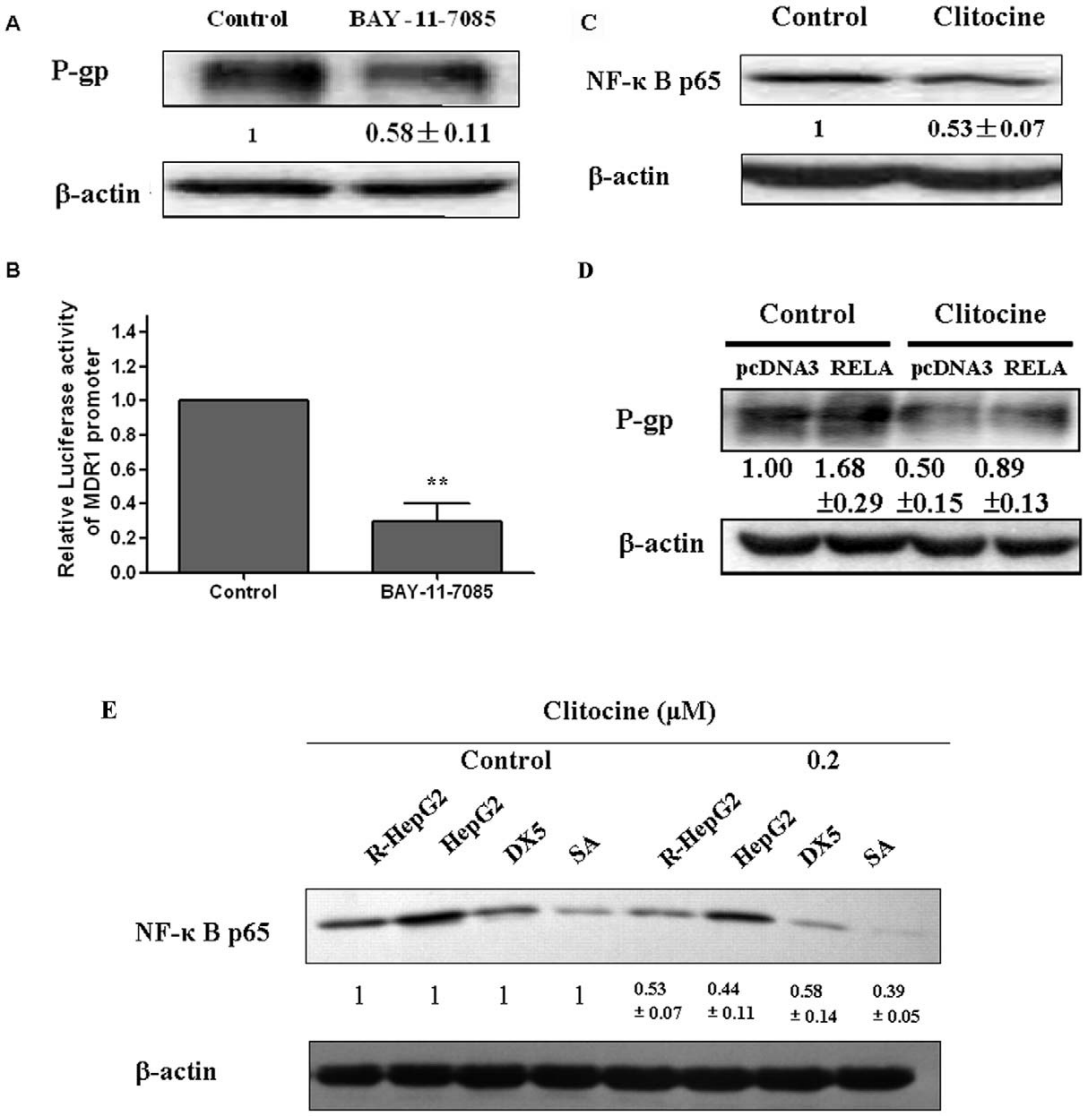
NF-κB mediated the clitocine suppression on MDR1. (A) P-gp in R-HepG2 cells with BAY-11-7085 (10 µM) treatment was assessed by Western blot assay. After treatment with 10 µM BAY-11-7085 for 48 h, P-gp protein level in R-HepG2 cells was measured by Western blot analysis. (B) The relative luciferase activity of reporter carrying the full length MDR1 promoter was determined by Dual-Luciferase Reporter Assay System. R-HepG2 cells were transiently transfected with full length MDR1 promoter reporter followed by treatment with 10 µM BAY-11-7085 for 24 h. After that the cells were lysed and the luciferase activity measured. Data are mean ± SD, *N* = 3. ^**^ *P*<0.01 vs. control. (C) After treatment with 0.2 µM clitocine for 48 h, Western blot assay was performed in R-HepG2 cells. (D) R-HepG2 cells were transiently transfected with pcDNA3-RELA and empty pcDNA3 (negative control) followed by treatment with 0.2 µM clitocine for 48 h. P-gp protein level was measured by western blot analysis. Data are mean ± SD, *N* = 3. (E) After treatment with 0.2 µM clitocine for 48 h, Western blot assay was performed in R-HepG2, HepG2, MES-SA and MES-SA/Dx5 cells to measure the NF-κB p65 level.

To elucidate the ways that NF-κB mediates the clitocine suppression on MDR1, the functional role of NF-κB in MDR1 expression was first examined. R-HepG2 cells were treated with NF-κB inhibitor BAY-11-7085 for 48 h followed by Western blot analysis. As shown in Fig. 4A, BAY-11-7085 treatment suppressed the expression of P-gp and also the activity of reporter with full length MDR1 promoter (Fig. 4B), indicating that NF-κB will act as a transcription activator of MDR1.

To confirm whether NF-κB is involved in the suppression of MDR1 by clitocine, the effect of clitocine on NF-κB expression was examined. As shown in Fig. 4C, clitocine treatment could only inhibit the expression of NF-κB p65 but not other NF-κB members such as NF-κB p50. Furthermore, transfection with vector carrying NF-κB p65 increased the level of P-gp and also counteracted the clitocine effect on P-gp in R-HepG2 cells (Fig. 4D). More interestingly, clitocine can also effectively suppress the expression of NF-κB p65 in HepG2, MES-SA and MES-SA/Dx5 cells (Fig. 4E).

### Inhibitory effect of clitocine on XIAP expression and NF-κB activation

**Figure 5:**
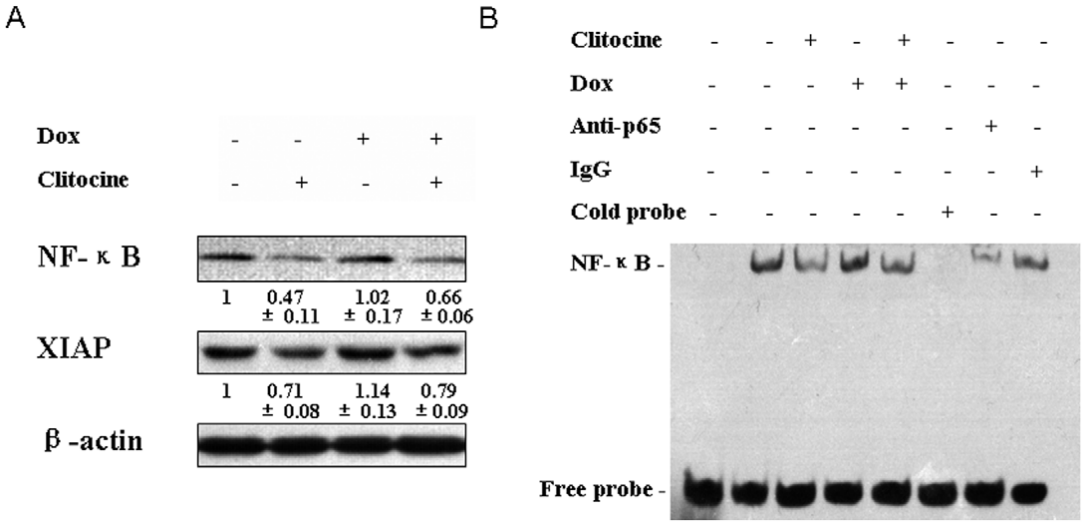
Clitocine inhibits the expression of XIAP and activation of NF-κB in R-HepG2 cells. (A) After treatment with 0.2 µM clitocine, 1.2 µM doxorubicin or combined agents for 48 h, Western blot was performed to assess the expression of NF-κB p65 and its' target mediator XIAP. (B) After treatment with 0.2 µM clitocine, 1.2 µM doxorubicin or combined for 60 min, nuclear protein was extract and analyzed using ESMA with a double-stranded oligo nucleotide probe containing NF-kB consensus sequence. Excess molar ratio of cold probe (lane 6), anti-p65 subunit of NF-kB antibody (lane 7) and IgG (lane 8) were used for the specificity assay.

It was reported that NF-κB was involved in regulation of a large number of genes that control cell proliferation, cell survival and immune responses [32]. At the present, we also examined the important downstream targets of NF-κB such as Inhibitors of apoptosis (IAPs) by Western blot assay. As shown in Fig. 5A, doxorubicin treatment could up-regulate NF-κB level while this effect can be reversed by clitocine; clitocine alone or in combination with doxorubicin significantly down-regulate the expression of NF-κB. Similar result was also observed that doxorubicin activated the XIAP expression while clitocine alone or in combination with doxorubicin suppressed the XIAP expression (Fig. 5A).

It was well known NF-κB may enter into nucleus under multiple stimuli to activate its' target genes [33]. Electrophoresis Mobility Shift Assay (EMSA) was chosen to verify the inhibitory effect of clitocine on NF-κB activation. The data of EMSA indicated that R-HepG2 cells exhibited spontaneously activated NF-kB activity while clitocine could rapidly inactivated NF-kB and its' nuclear translocation significantly (Fig. 5B). However, doxorubicin treatment induced the activation of NF-kB (Fig. 5B). More interestingly, it looked like that clitocine could effectively overcome the activation of NF-κB resulted by doxorubicin treatment (Fig. 5B) and same trend can be seen in the XIAP expression (Fig. 5A).

### NF-κB and P-gp are tightly regulated by clitocine in R-HepG2 cells and in tumor tissues

**Figure 6:**
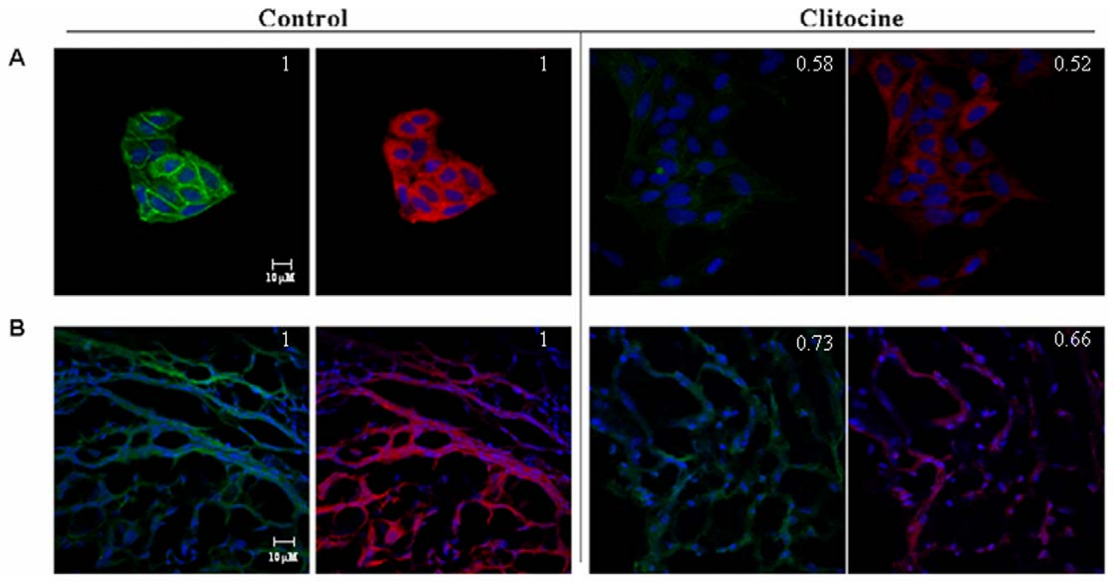
Clitocine inhibits the expressions of NF-κB p65 and P-gp in R-HepG2 cells *in vitro* and *in vivo* tumor tissue from nude mice. (A) After treatment with 0.2 µM clitocine for 48 h, immunocytochemistry assay was perpormed in R-HepG2 cells using antibodies: NF-κB p65 and P-gp. (B) After intravenous injection of clitocine (10 mg/kg, once a day for one week), tumors were excised from tumor bearing animals. Tumors were fixed with 4% paraformaldehyde in PBS and cut into 6 µm frozen sections and immunocytochemistry assay was performed. The expression levels of these two proteins were observed with a LSM 510 fluorescence microscope fitted with appropriate filters and the images captured with an Orca II CCD camera (Hamamatsu, Bridgewater, NJ). The pixel intensity was analyzed with Image J. A representative experiment was shown.

Animal experiment revealed that clitocine treatment could significantly inhibit the growth of human hepatocellular carcinoma HepG2 and R-HepG2 tumor (data not shown). The effect of clitocine on the expressions of NF-κB and P-gp in R-HepG2 cells *in vitro* and tumour tissue *in vivo* was examined by immunohistochemistry. As shown in Fig. 6A, the expressions of NF-κB p65 and P-gp were both suppressed by clitocine, showing a positive relationship between these two proteins in R-HepG2 cells upon clitocine treatment. Similar results were observed in R-HepG2 tumor tissue from nude mice with clitocine treatment (Fig. 6B). The pixel intensity was analyzed with Image J (NIH) and the data was normalized with signal of DAPI. The relative value was labeled at the upper right corner of the figures.

## Discussion

Development of MDR reflects not only the multiple genetical and epigenetical changes occuring inside the cells under cytotoxic conditions, but also a normal physiological response of cells to struggle for survival. A great number of studies have been carried out over the last 3 decades to understand the pharmacological and toxicological effect of ABC efflux transporters. Among them, the P-gp is an important membrane transporter that has been recognized as the most vital barrier to effective drug delivery and plays a key role in the development of MDR. An attractive strategy to improve the drug delivery and overcome drug resistance is inhibition of the efflux pump P-gp transporter. The aim of this study was to find a more effective MDR-reversing compound and get insight into its underlying molecular mechanism. In the present study, we demonstrated that clitocine, a nucleoside extracted from *Leucopaxillus giganteus* can circumvent MDR in drug resistant R-HepG2 and MES-SA/Dx5 cells by suppressing the P-gp expression.

R-HepG2 and MES-SA/Dx5 cells showed over-expression of P-gp and clitocine could down-regulate P-gp expression in both cell lines (Fig. 2A). However, it seemed that the P-gp level in R-HepG2 cells was much higher than that in MES-SA/Dx5 cells and clitocine exerted more effective regulatory activity in the former (Fig. 2A). R-HepG2 and MES-SA/Dx5 cells presented much lower sensitivity to doxorubicin compared with parental cells (data not shown). Interestingly, the compound clitocine was found to effectively enhance the anticancer activity of doxorubicin in drug resistant cells at the dose of ≥0.2 µM (Fig. 2C and E). Here we chose R-HepG2 cells for the further research on clitocine's inhibitory effect in P-gp expression. It was observed clitocine also increased the doxorubicin accumulation in R-HepG2 cells (Fig. 2F), suggesting that the P-gp related pump may be inhibited by clitocine. The hypothesis was supported as P-gp was significantly decreased at both mRNA and protein levels in R-HepG2 cells under clitocine treatment (Fig. 2A and G). Subsequently, ciltocine was found to remarkably inhibit the activity of MDR1 gene promoter, indicating that the effect of clitocine on MDR1 is at the transcriptional level (Fig. 2H).

The proximal promoter region of the human MDR1 gene was firstly identified in 1987. The promoter contains a consensus CAAT box and two GC box-like sequences while lacks many sequence elements associated with more active cellular and viral promoter, such as canonical TATA or CCAAT sequences upstream of initial site [34]. To get more in-depth into the mechanism for transcriptional regulation of clitocine on MDR1, a series of 5′-end truncated MDR1 promoter constructs were cloned and their transcriptional activities under the clitocine treatment were examined. The results suggested that the fragment −450 to −193 was indispensable in the suppression effect of clitocine on MDR1 (Fig. 3A). By computer-based sequence analysis, a single consensus binding site (GAAAATTTCC at −324 to −315) for transcription factor NF-κB was identified in the region from −450 to − 193 (Fig. 3B). Mutation in the NF-κB putative binding motif (−324 to −315 bp) could significantly reverse the inhibitory effect of clitocine on the activity of reporter with full length MDR1 promoter (Fig. 3C). Furthermore, the interaction of NF-κB with the region was confirmed by chromatin immunoprecipitaition (CHIP) assay (Fig. 3D). The results therefore supported that NF-κB may mediate the clitocine suppression on MDR1.

NF-κB is a family of ubiquitous transcription factors which can be activated by a large number of stimuli such as cytokines or DNA damaging agents including chemotherapeutic drugs [33], [35]. Normally, NF-κB members are kept inactive in the cytoplasm via binding with inhibitory moleculaes of IκB family. In response to multiple stimuli, the IκB molecules become phosphorylated on two critical serine residues by IKK; free NF-κB enters into the nucleus and activates transcription of a variety of genes participating in the immune and inflammatory response, cell adhesion, growth control, and protection against apoptosis [36], [37], [38], [39]. NF-κB is the central regulator of immune responses through inducing various genes; some of them act as potential inhibitors of apoptosis especially cellular inhibitors of apoptosis (c-IAPs) [32]. Several studies indicated that NF-κB inhibition could increase cellular response to cytotoxic agents [40], [41], [42], [43], but this activity was probably dependent on the cell lines, the agents used and the extent of NF-κB suppression [44], [45]. The activation of NF-κB signaling could up-regulate MDR1 gene expression in human hepatoma cells [46]. Moreover, NF-κB might directly bind the MDR 1 gene promoter and induce drug resistance through MDR1 over-expression in HCT15 colon cancer cells [47]. To confirm the relationship between NF-κB and P-gp expression in R-HepG2 cells, an NF-κB inhibitor, Bay 11-7082 was used. Bay 11-7082 remarkably down-regulated both the P-gp protein level and transcriptional activity of full length MDR1 promoter in R-HepG2 cells (Fig. 4A and B). These results suggested that NF-κB may be involved in the regulation of MDR1 in R-HepG2 cells. Furthermore, NF-κB p65 was down-regulated under clitocine treatment in R-HepG2 cells (Fig. 4C) while no significant decrease was seen for NF-κB p50 (data not show). Interestingly, NF-κB p65 over-expression can reverse the inhibitory effect of clitocine on P-gp (Fig. 4D). The regulatory effect of citocine on NF-κB p65 expression in other cell lines was also measured by western blot analysis. As shown in Fig. 4E clitocine can also down-regulate the NF-κB p65 expression in other cell lines including MES-SA/Dx5 and their parental HepG2 and MES-SA cells. Furthermore, clitocine could also suppress the expression of NF-κB and its' downstream genes such as XIAP even in presence of doxorubicin in RD cells (Fig. 5A). More importantly, clitocine significantly overcome the activation of NF-κB by doxorubicin which was characterized as nuclear translocation of NF-κB (Fig. 5B). By immunocytochemistry assay, the expressions of NF-κB p65 and P-gp were found to be tightly coupled in R-HepG2 cells and both could be suppressed by cltiocine *in vitro* and *in vivo* (Fig. 5). These data indicated that NF-κB p65 was the target via which clitocine could transcriptionally regulate MDR1 in R-HepG2 and maybe in MES-SA/Dx5 cells.

In conclusion, our data strongly support that clitocine can suppress P-glycoprotein over-expression in doxorubicin induced multidrug resistant R-HepG2 and MES-SA/Dx5 cells via inhibition of NF-κB p65 protein level and it's activation. Therefore, clitocine could be a potent NF-κB p65 inhibitor for cancer treatment as sensitizers to anticancer drugs [48] and have potential therapeutic application in cancer and inflammatory diseases.

## Supporting Information

Figure S1 The HPLC analysis of clitocine.
(TIF)

Figure S2 Synergy of combination of clitocine and doxorubicin in the proliferation of R-HepG2 cells. The MTT data was analyzed by Chou-Talalay method, (combination index >1 indicates antagonism, = 1 indicates additivity, and <1 indicates synergy).
(TIF)

Table S1 The NMR data of clitocine.
(TIF)

## References

[1] Gottesman MM, Pastan I (1993) Biochemistry of multidrug resistance mediated by the multidrug transporter. Annu Rev Biochem 62: 385–427. 8102521 10.1146/annurev.bi.62.070193.002125

[2] Ambudkar SV, Dey S, Hrycyna CA, Ramachandra M, Pastan I, et al (1999) Biochemical, cellular, and pharmacological aspects of the multidrug transporter. Annu Rev Pharmacol Toxicol 39: 361–398. 10331089 10.1146/annurev.pharmtox.39.1.361

[3] Ling V, Kartner N, Sudo T, Siminovitch L, Riordan JR (1983) Multidrug-resistance phenotype in Chinese hamster ovary cells. Cancer Treat Rep 67: 869–874. 6354434

[4] Bosch I, Croop J (1996) P-glycoprotein multidrug resistance and cancer. Biochim Biophys Acta 1288: F37–54. 8876632 10.1016/0304-419x(96)00022-4

[5] Aller SG, Yu J, Ward A, Weng Y, Chittaboina S, et al (2009) Structure of P-glycoprotein reveals a molecular basis for poly-specific drug binding. Science 323: 1718–1722. 19325113 10.1126/science.1168750 PMC2720052

[6] Hennessy M, Spiers JP (2007) A primer on the mechanics of P-glycoprotein the multidrug transporter. Pharmacol Res 55: 1–15. 17095241 10.1016/j.phrs.2006.10.007

[7] Krishna R, Mayer LD (2000) Multidrug resistance (MDR) in cancer. Mechanisms, reversal using modulators of MDR and the role of MDR modulators in influencing the pharmacokinetics of anticancer drugs. Eur J Pharm Sci 11: 265–283. 11033070 10.1016/s0928-0987(00)00114-7

[8] Yusa K, Tsuruo T (1989) Reversal mechanism of multidrug resistance by verapamil: direct binding of verapamil to P-glycoprotein on specific sites and transport of verapamil outward across the plasma membrane of K562/ADM cells. Cancer Res 49: 5002–5006. 2569930

[9] Loo TW, Clarke DM (2001) Defining the drug-binding site in the human multidrug resistance P-glycoprotein using a methanethiosulfonate analog of verapamil, MTS-verapamil. J Biol Chem 276: 14972–14979. 11279063 10.1074/jbc.M100407200

[10] Slater L, Sweet P, Wetzel M, Stupecky M, Osann K (1995) Comparison of cyclosporin A, verapamil, PSC-833 and cremophor EL as enhancing agents of VP-16 in murine lymphoid leukemias. Leuk Res 19: 543–548. 7658700 10.1016/0145-2126(95)00029-n

[11] Chin KV, Ueda K, Pastan I, Gottesman MM (1992) Modulation of activity of the promoter of the human MDR1 gene by Ras and p53. Science 255: 459–462. 1346476 10.1126/science.1346476

[12] Cornwell MM, Smith DE (1993) SP1 activates the MDR1 promoter through one of two distinct G-rich regions that modulate promoter activity. J Biol Chem 268: 19505–19511. 8103518

[13] Zhou G, Kuo MT (1998) Wild-type p53-mediated induction of rat mdr1b expression by the anticancer drug daunorubicin. J Biol Chem 273: 15387–15394. 9624121 10.1074/jbc.273.25.15387

[14] Zhou G, Kuo MT (1997) NF-kappaB-mediated induction of mdr1b expression by insulin in rat hepatoma cells. J Biol Chem 272: 15174–15183. 9182539 10.1074/jbc.272.24.15174

[15] Fine RL, Chambers TC, Sachs CW (1996) P-glycoprotein, multidrug resistance and protein kinase C. Stem Cells 14: 47–55. 8820951 10.1002/stem.140047

[16] Tang XY, Zhu YQ (2008) Epigallocatechin-3-gallate suppressed the over-expression of HSP 70 and MDR1 induced by heat shock in SGC 7901. J Chemother 20: 355–360. 18606592 10.1179/joc.2008.20.3.355

[17] Chin KV, Tanaka S, Darlington G, Pastan I, Gottesman MM (1990) Heat shock and arsenite increase expression of the multidrug resistance (MDR1) gene in human renal carcinoma cells. J Biol Chem 265: 221–226. 1967174

[18] Fujita T, Ito K, Izumi H, Kimura M, Sano M, et al (2005) Increased nuclear localization of transcription factor Y-box binding protein 1 accompanied by up-regulation of P-glycoprotein in breast cancer pretreated with paclitaxel. Clin Cancer Res 11: 8837–8844. 16361573 10.1158/1078-0432.CCR-05-0945

[19] David GL, Yegnasubramanian S, Kumar A, Marchi VL, De Marzo AM, et al (2004) MDR1 promoter hypermethylation in MCF-7 human breast cancer cells: changes in chromatin structure induced by treatment with 5-Aza-cytidine. Cancer Biol Ther 3: 540–548. 15034303 10.4161/cbt.3.6.845

[20] Yatouji S, El-Khoury V, Trentesaux C, Trussardi-Regnier A, Benabid R, et al (2007) Differential modulation of nuclear texture, histone acetylation, and MDR1 gene expression in human drug-sensitive and -resistant OV1 cell lines. Int J Oncol 30: 1003–1009. 17332941

[21] Kim SN, Kim NH, Lee W, Seo DW, Kim YK (2009) Histone deacetylase inhibitor induction of P-glycoprotein transcription requires both histone deacetylase 1 dissociation and recruitment of CAAT/enhancer binding protein beta and pCAF to the promoter region. Mol Cancer Res 7: 735–744. 19435809 10.1158/1541-7786.MCR-08-0296

[22] Chung SY, Sung MK, Kim NH, Jang JO, Go EJ, et al (2005) Inhibition of P-glycoprotein by natural products in human breast cancer cells. Arch Pharm Res 28: 823–828. 16114498 10.1007/BF02977349

[23] Mothana RA, Lindequist U, Gruenert R, Bednarski PJ (2009) Studies of the in vitro anticancer, antimicrobial and antioxidant potentials of selected Yemeni medicinal plants from the island Soqotra. BMC Complement Altern Med 9: 7. 19320966 10.1186/1472-6882-9-7 PMC2667473

[24] Patanasethanont D, Nagai J, Matsuura C, Fukui K, Sutthanut K, et al (2007) Modulation of function of multidrug resistance associated-proteins by Kaempferia parviflora extracts and their components. Eur J Pharmacol 566: 67–74. 17481606 10.1016/j.ejphar.2007.04.001

[25] Moss RJ, Petrie CR, Meyer RB Jr, Nord LD, Willis RC, et al (1988) Synthesis, intramolecular hydrogen bonding, and biochemical studies of clitocine, a naturally occurring exocyclic amino nucleoside. J Med Chem 31: 786–790. 3351857 10.1021/jm00399a017

[26] Lee CH, Daanen JF, Jiang M, Yu H, Kohlhaas KL, et al (2001) Synthesis and biological evaluation of clitocine analogues as adenosine kinase inhibitors. Bioorg Med Chem Lett 11: 2419–2422. 11549437 10.1016/s0960-894x(01)00454-1

[27] Fortin H, Tomasi S, Delcros JG, Bansard JY, Boustie J (2006) In vivo antitumor activity of clitocine, an exocyclic amino nucleoside isolated from Lepista inversa. ChemMedChem 1: 189–196. 16892351 10.1002/cmdc.200500029

[28] Ren G, Zhao YP, Yang L, Fu CX (2008) Anti-proliferative effect of clitocine from the mushroom Leucopaxillus giganteus on human cervical cancer HeLa cells by inducing apoptosis. Cancer Lett 262: 190–200. 18222036 10.1016/j.canlet.2007.12.013

[29] Liu F, Hu X, Zimmerman M, Waller JL, Wu P, et al (2011) TNFalpha cooperates with IFN-gamma to repress Bcl-xL expression to sensitize metastatic colon carcinoma cells to TRAIL-mediated apoptosis. PLoS One 6: e16241. 21264227 10.1371/journal.pone.0016241 PMC3022032

[30] Tang PM, Chan JY, Zhang DM, Au SW, Fong WP, et al (2007) Pheophorbide a, an active component in Scutellaria barbata, reverses P-glycoprotein-mediated multidrug resistance on a human hepatoma cell line R-HepG2. Cancer Biol Ther 6: 504–509. 17457045 10.4161/cbt.6.4.3814

[31] Chou TC (2010) Drug combination studies and their synergy quantification using the Chou-Talalay method. Cancer Res 70: 440–446. 20068163 10.1158/0008-5472.CAN-09-1947

[32] Karin M, Lin A (2002) NF-kappaB at the crossroads of life and death. Nat Immunol 3: 221–227. 11875461 10.1038/ni0302-221

[33] Pahl HL (1999) Activators and target genes of Rel/NF-kappaB transcription factors. Oncogene 18: 6853–6866. 10602461 10.1038/sj.onc.1203239

[34] Ueda K, Pastan I, Gottesman MM (1987) Isolation and sequence of the promoter region of the human multidrug-resistance (P-glycoprotein) gene. J Biol Chem 262: 17432–17436. 2891692

[35] Karin M (1999) How NF-kappaB is activated: the role of the IkappaB kinase (IKK) complex. Oncogene 18: 6867–6874. 10602462 10.1038/sj.onc.1203219

[36] Auphan N, DiDonato JA, Rosette C, Helmberg A, Karin M (1995) Immunosuppression by Glucocorticoids: Inhibition of NF-κB Activity Through Induction of IκB Synthesis. Science 270: 286–290. 7569976 10.1126/science.270.5234.286

[37] DiDonato JA, Hayakawa M, Rothwarf DM, Zandi E, Karin M (1997) A cytokine-responsive IkappaB kinase that activates the transcription factor NF-kappaB. Nature 388: 548–554. 9252186 10.1038/41493

[38] Chu ZL, Shin YA, Yang JM, DiDonato JA, Ballard DW (1999) IKKgamma mediates the interaction of cellular IkappaB kinases with the tax transforming protein of human T cell leukemia virus type 1. J Biol Chem 274: 15297–15300. 10336413 10.1074/jbc.274.22.15297

[39] Israel A (2010) The IKK complex, a central regulator of NF-kappaB activation. Cold Spring Harb Perspect Biol 2: a000158. 20300203 10.1101/cshperspect.a000158 PMC2829958

[40] Wang CY, Mayo MW, Baldwin AS Jr (1996) TNF- and cancer therapy-induced apoptosis: potentiation by inhibition of NF-kappaB. Science 274: 784–787. 8864119 10.1126/science.274.5288.784

[41] Aydin C, Sanlioglu AD, Bisgin A, Yoldas B, Dertsiz L, et al (2010) NF-kappaB targeting by way of IKK inhibition sensitizes lung cancer cells to adenovirus delivery of TRAIL. BMC Cancer 10: 584. 20977779 10.1186/1471-2407-10-584 PMC2988028

[42] Wang CY, Cusack JC Jr, Liu R, Baldwin AS Jr (1999) Control of inducible chemoresistance: enhanced anti-tumor therapy through increased apoptosis by inhibition of NF-kappaB. Nat Med 5: 412–417. 10202930 10.1038/7410

[43] Bauer JA, Lupica JA, Schmidt H, Morrison BH, Haney RM, et al (2007) Nitrosylcobalamin potentiates the anti-neoplastic effects of chemotherapeutic agents via suppression of survival signaling. PLoS One 2: e1313. 18074035 10.1371/journal.pone.0001313 PMC2117345

[44] Bentires-Alj M, Hellin AC, Ameyar M, Chouaib S, Merville MP, et al (1999) Stable inhibition of nuclear factor kappaB in cancer cells does not increase sensitivity to cytotoxic drugs. Cancer Res 59: 811–815. 10029068

[45] Kasibhatla S, Brunner T, Genestier L, Echeverri F, Mahboubi A, et al (1998) DNA damaging agents induce expression of Fas ligand and subsequent apoptosis in T lymphocytes via the activation of NF-kappa B and AP-1. Mol Cell 1: 543–551. 9660938 10.1016/s1097-2765(00)80054-4

[46] Kuo MT, Liu Z, Wei Y, Lin-Lee YC, Tatebe S, et al (2002) Induction of human MDR1 gene expression by 2-acetylaminofluorene is mediated by effectors of the phosphoinositide 3-kinase pathway that activate NF-kappaB signaling. Oncogene 21: 1945–1954. 11960367 10.1038/sj.onc.1205117

[47] Bentires-Alj M, Barbu V, Fillet M, Chariot A, Relic B, et al (2003) NF-kappaB transcription factor induces drug resistance through MDR1 expression in cancer cells. Oncogene 22: 90–97. 12527911 10.1038/sj.onc.1206056

[48] Nakanishi C, Toi M (2005) Nuclear factor-kappaB inhibitors as sensitizers to anticancer drugs. Nat Rev Cancer 5: 297–309. 15803156 10.1038/nrc1588

