# Supplementary figures and images for: Clitocine Reversal of P-Glycoprotein Associated Multi-Drug Resistance through Down-Regulation of Transcription Factor NF-κB in R-HepG2 Cell Line
Source: PLoS One. 2012 Aug 22;7(8):e40720. doi: 10.1371/journal.pone.0040720 (PMC3425549; doi:10.1371/journal.pone.0040720)

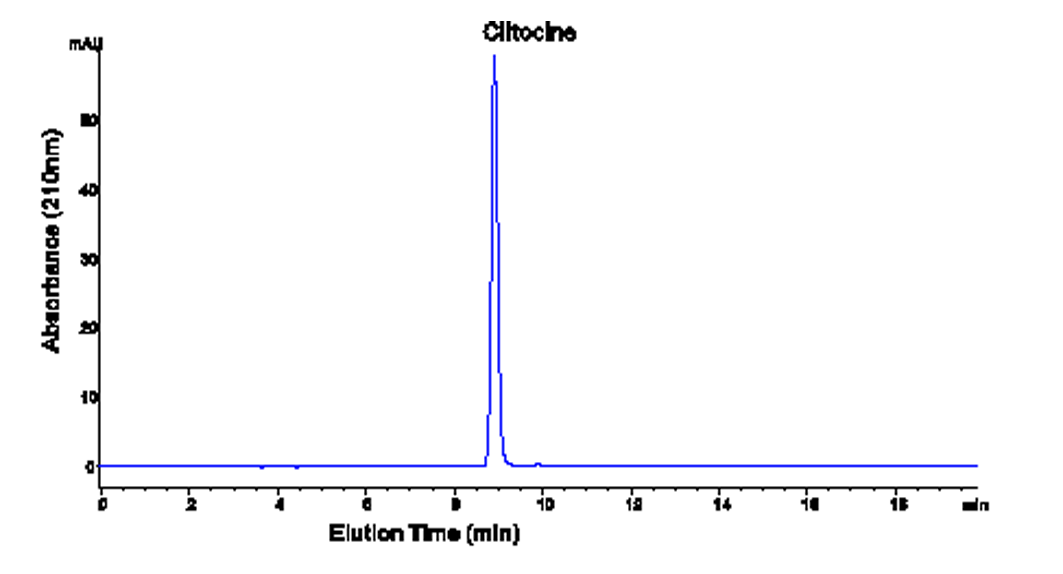

Supplement: Figure S1 — The HPLC analysis of clitocine. (TIF) [file pone.0040720.s001.tif]

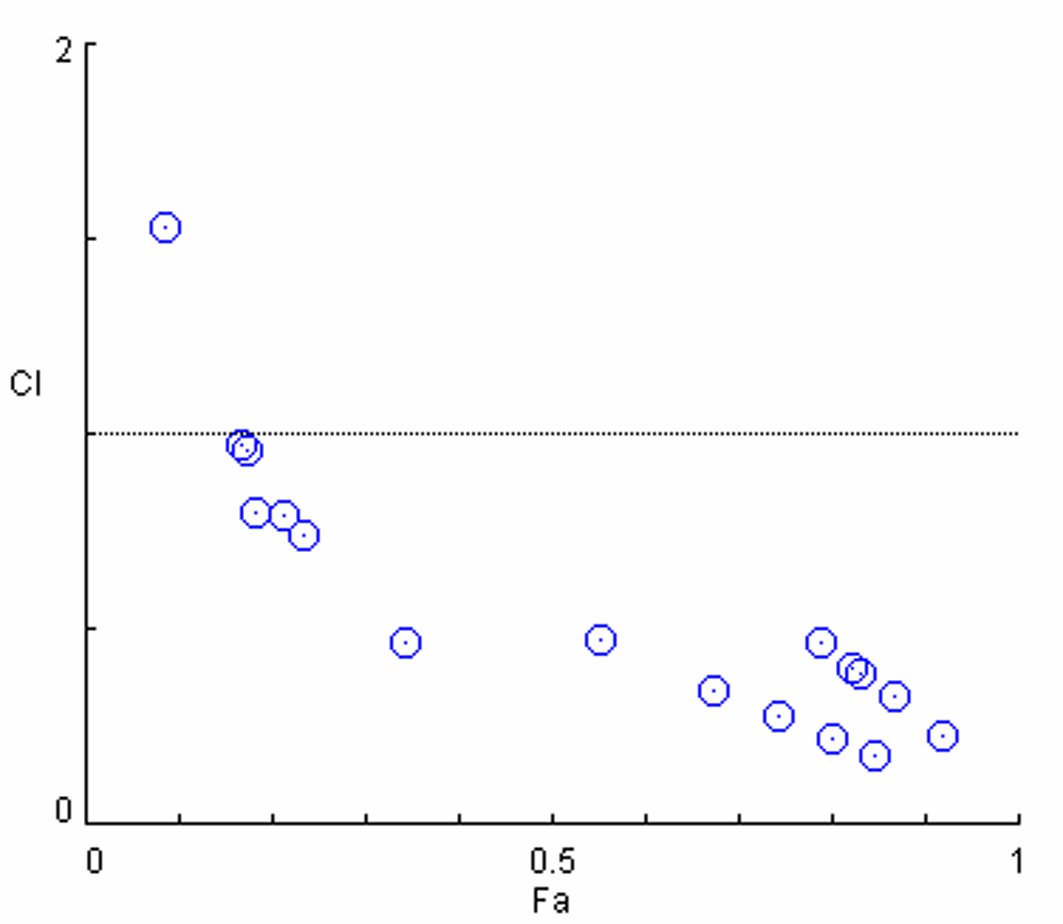

Supplement: Figure S2 — Synergy of combination of clitocine and doxorubicin in the proliferation of R-HepG2 cells. The MTT data was analyzed by Chou-Talalay method, (combination index >1 indicates antagonism, = 1 indicates additivity, and <1 indicates synergy). (TIF) [file pone.0040720.s002.tif]

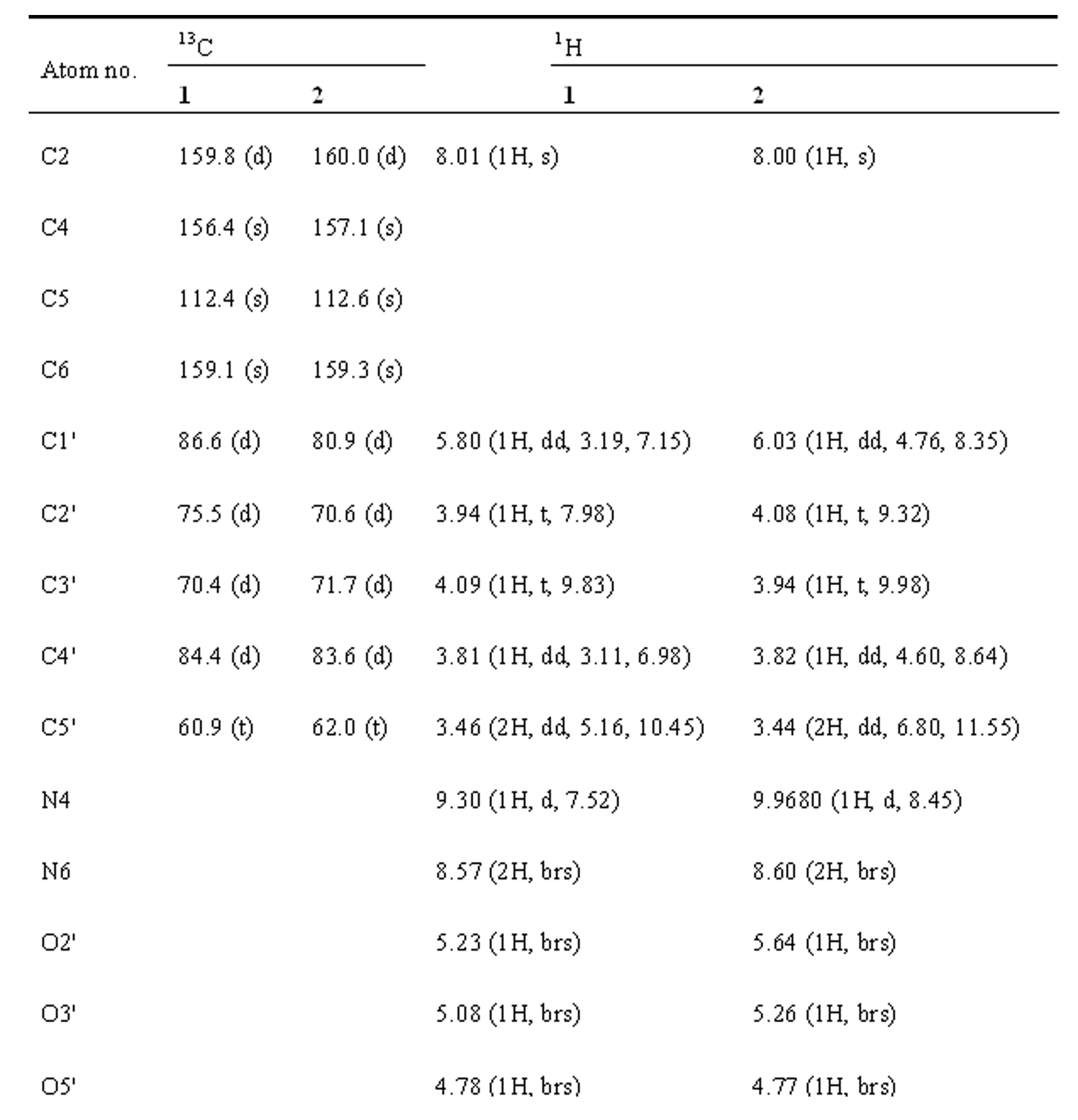

Supplement: Table S1 — The NMR data of clitocine. (TIF) [file pone.0040720.s003.tif]
